# Alpha-synuclein aggresomes inhibit ciliogenesis and multiple functions of the centrosome

**DOI:** 10.1242/bio.054338

**Published:** 2020-10-05

**Authors:** Anila Iqbal, Marta Baldrighi, Jennifer N. Murdoch, Angeleen Fleming, Christopher J. Wilkinson

**Affiliations:** 1 Centre for Biomedical Sciences, Department of Biological Sciences, Royal Holloway University of London, Egham, Surrey, TW20 0EX, UK; 2 Department for Physiology, Development and Neuroscience, University of Cambridge, Cambridge, CB2 3DY, UK

**Keywords:** Parkinson's disease, Centrosome, Aggresome, Cilia, Lewy body, Alpha-synuclein

## Abstract

Protein aggregates are the pathogenic hallmarks of many different neurodegenerative diseases and include the accumulation of α-synuclein, the main component of Lewy bodies found in Parkinson's disease. Aggresomes are closely-related, cellular accumulations of misfolded proteins. They develop in a juxtanuclear position, adjacent to the centrosome, the microtubule organizing centre of the cell, and share some protein components. Despite the long-standing observation that aggresomes/Lewy bodies and the centrosome sit side-by-side in the cell, no studies have been done to see whether these protein accumulations impede organelle function. We investigated whether the formation of aggresomes affected key centrosome functions: its ability to organise the microtubule network and to promote cilia formation. We find that when aggresomes are present, neuronal cells are unable to organise their microtubule network. New microtubules are not nucleated and extended, and the cells fail to respond to polarity cues. Since neurons are polarised, ensuring correct localisation of organelles and the effective intracellular transport of neurotransmitter vesicles, loss of centrosome activity could contribute to functional deficits and neuronal cell death in Parkinson's disease. In addition, we provide evidence that many cell types, including dopaminergic neurons, cannot form cilia when aggresomes are present, which would affect their ability to receive extracellular signals.

## Abstract (precis)

**Summary:** Aggresomes of alpha-synuclein – the main component of the Lewy bodies in Parkinson's disease – prevent microtubule nucleation by the centrosome and inhibit cells, including olfactory neurons, from making cilia.

## INTRODUCTION

Parkinson's disease (PD) is a progressive neurodegenerative condition that affects 1 in 500 of the population (Schrag et al., 2000; Alves et al., 2008). Dopaminergic neurons in the substantia nigra pars compacta are first affected (Alves et al., 2008). As the disease progresses, other parts of the brain and nervous system are affected, with dementia occurring in later stages. Within neurons of PD patients, large α-synuclein-positive intracellular inclusions known as Lewy bodies are observed (Wakabayashi et al., 2012). Increased α-synuclein (α-syn) levels, which occur in rare cases with multiplication of the SNCA gene, are sufficient to cause PD (Singleton et al., 2003; Ibanez et al., 2004) and mutations associated with SNCA lead to an increase in aggregation propensity (Conway et al., 1998; Polymeropoulos et al., 1997; Kruger et al., 1998). These mutations cause α-syn to form oligomers, fibrils then aggregates. Large aggregates of α-syn constitute the Lewy bodies frequently found in the neurons of PD patients (Baba et al., 1998; Spillantini et al., 1997). It is unclear if these aggregates or Lewy bodies protect the cell from smaller unfolded units of α-syn or if these structures are pathogenic and ultimately contribute to neuronal death.

Lewy bodies are observed in several diseases [PD, dementia with Lewy bodies, incidental Lewy body disease (Wakabayashi et al., 2012)] but are not found in healthy cells and they are related to the aggresome, a structure found in cells that are processing large amounts of waste, unfolded polypeptide (Johnston et al., 1998). The aggresome is a juxtanuclear inclusion body containing heat-shock proteins and components of the ubiquitin-proteasome system (Olzmann et al., 2008). These components are also found within Lewy bodies and they share ultrastructural similarities. This has led to the proposal that Lewy bodies are derived from aggresomes to specifically deal with misfolded α-syn (McNaught et al., 2002; Olanow et al., 2004).

The juxtanuclear location of the aggresome is shared by the centrosome, the microtubule organising centre of the cell. Indeed, they sit side-by-side and centrosomal markers such as γ-tubulin are often used to detect the aggresome, alongside the intermediate filaments such as vimentin that cage the structure (McNaught et al., 2002). While the position of the aggresome at the centre of the microtubule network has logic in terms of transporting unfolded protein to a central location to be further processed, it may affect the function of the centrosome, whose major role is to organise this part of the cytoskeleton. Steric hindrance on a macromolecular scale may perturb centrosome function. Furthermore, the centrosome is used to make the cilium. This hair-like structure on the surface of cells has important roles both in motility, inter-cellular communication and monitoring the external environment, with specialised cilia housing photopigments and olfactory receptors (Nigg and Raff, 2009; Ibanez-Tallon et al., 2003). To make the cilium, the centrioles of the centrosome migrate to the cell surface, a process that could be blocked by a large aggregate of protein smothering the centrosome or sticking it to the nucleus.

The colocalization of the aggresome and centrosome and the sharing of protein components has been known for nearly 20 years. Generating aggresomes by proteasome inhibition via MG132 treatment inhibits microtubule-based transport of lysosomes and mitochondria near to the centrosome generating an ‘entrapment zone’ (Zaarur et al., 2014). Furthermore, such aggresomes perturb microtubule nucleation by the centrosome and disrupt transport of the dopamine transporter in a neuronal cell line (Diaz-Corrales et al., 2012). However, the disease relevance of these aggresomes is unclear and the effect of α-syn-positive aggresomes on the function of the centrosome has not been investigated. This could have important implications for the aetiology of PD and other neurodegenerative diseases in which such aggregates are formed. In this study we sought to test whether multiple functions of the centrosome were impeded by the presence of α-syn-positive aggresomes in their close vicinity using both *in vitro* and *in vivo* models. Our results suggest that inhibition of centrosome function might contribute to loss of function in neurons where there is aggregation of α-syn.

## RESULTS

### Aggresomes localise in close proximity to the centrosome

We sought to make aggresomes in the cells on which we performed subsequent experiments by two parallel means (Tanaka et al., 2004; Winslow et al., 2010). Cells were transfected with expression constructs encoding GFP fusions of human α-syn or mutant versions, A30P and A53T, found in familial cases of Parkinson's (Kruger et al., 1998; Polymeropoulos et al., 1997) or treated with MG132, a proteasome inhibitor. Our primary aim was to investigate the effect of α-syn-positive aggresomes on centrosome function and compare this to aggresomes generated via proteasome inhibition.

**Fig. 1.**
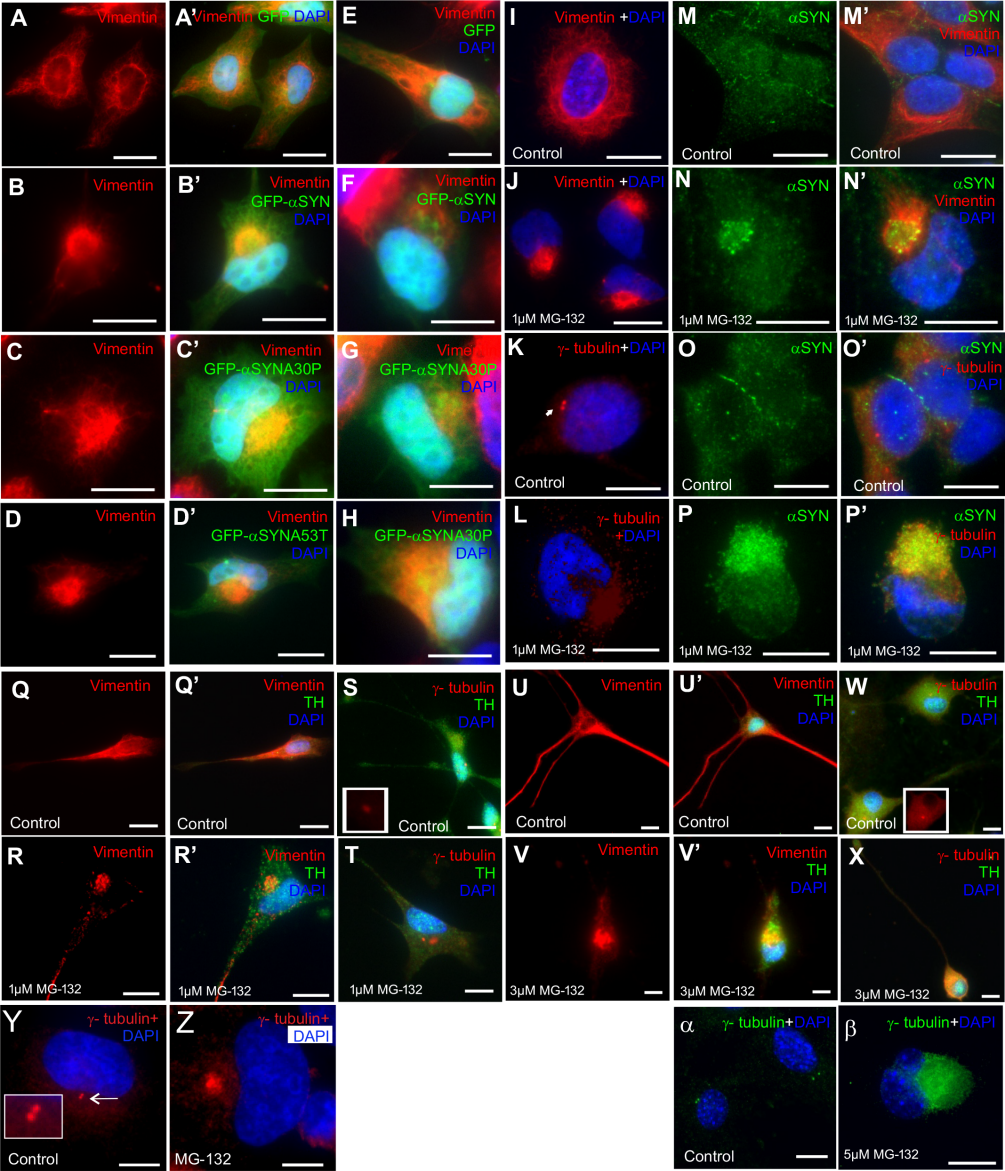
**Aggresomes can be formed in cells by overexpression of alpha synuclein or MG132 treatment.** (A,A′) Vimentin (red) in control HeLa cells forms a fibrous network around the nuclei (DAPI, blue), which is unaffected by the expression of the GFP. (B,B′) When expressing GFP- α-syn, vimentin-positive aggresomes appear in cells, juxtaposed to the nucleus. Similar results are obtained with GFP-α-synA30P (C,C′) and GFP-α-synA53T mutants (D,D′) and in SH-SY5Y cells treated in the same way (E–H). (I–L) Aggresomes can also be induced by treatment with MG132. (I,J) The vimentin distribution changes from a filamentous pattern around the nuclei to caging the aggresome. (K,L) γ-tubulin (red) staining the centrosomes as two punctae in control cells (K). Following MG132 treatment, γ-tubulin forms a condensed structure around the aggresome (L). (M–P) The expression pattern of endogenous α-syn was also investigated to determine whether this protein co-localises within the aggresome. (M,M′) In control cells, endogenous α-syn (green) staining was widespread and diffuse within the cytoplasm with vimentin (red) forming a filamentous network. (N,N′) Following MG132 treatment, α-syn aggregates were observed and co-localised with vimentin staining within the aggresomes. (O,O′) In control cells, γ-tubulin (red) was observed as two punctae with α-syn diffuse within the cytoplasm. (P,P′) γ-tubulin staining (red) also co-localises with endogenous α-syn in the aggresome when treated with MG132. (Q,Q′) Differentiated SH-SY5Y cells were mock-treated and vimentin (red) staining was observed surrounding the nuclei as well as along the axon. (R,R′) In cells treated with MG132 (1 μM for 18 h) vimentin staining changed to a compact structure near the nucleus, indicative of aggresomes. (S) In mock-treated cells, γ-tubulin formed two punctae next to the nucleus. (T) In MG132 treated cells, aggresomes were detected by γ-tubulin staining. Differentiated SH-SY5Y cells are TH positive (green). (U,U′) In rat basal ganglion neurons, vimentin staining (red) is abundant around the nuclei and along the axon. (V,V′) When treated with MG132, vimentin localises to the aggresome. (W) In rat basal ganglion neurons, the γ-tubulin is observed at two punctae close to the nucleus. (X) Upon MG132 treatment, the γ-tubulin staining now forms a larger structure next to the nucleus. (Y) In untreated RPE1-hTERT cells, γ-tubulin stains the centrosome. (Z) Upon MG132 treatment it stains the aggresome. (α,β) Similar results are obtained with MEFs. Scale bars: 10 μM. DNA/nuclei stained with DAPI (blue) where indicated.

The presence of aggresomes was then confirmed by staining with established markers for aggresomes: anti-vimentin or anti-γ-tubulin antibodies (Johnston et al., 1998; Olanow et al., 2004; Wigley et al., 1999). HeLa cells and SH-SY5Y cells, a neuroblastoma line, showed a dense zone of vimentin staining next to the nucleus, which was absent in untreated cells, whether α-syn transfection – native or mutant forms of the protein associated with familial use PD – or MG132 treatment was used to generate the aggresomes (Fig. 1A–J). Similar results were obtained with γ-tubulin staining, with a dense area of γ-tubulin staining instead of the usual two punctae representing the centrosome (Fig. 1K,L). Importantly, accumulation of endogenous α-syn was observed in aggresomes induced by MG132, demonstrating that both methods resulted in the accumulation of disease-associated, aggregate-prone proteins (Fig. 1M–P). It was notable that in treated cells, the centrosomes were frequently very difficult to detect by γ-tubulin alone, with the aggresomal gamma tubulin signal seemingly smothering that from the region around the centrioles.

SH-SY5Y cells can be differentiated into dopaminergic neurons if treated with retinoic acid. Since dopaminergic neurons are the first neurons to be affected in PD, we repeated the above experiment with differentiated SH-SY5Y cells and rat basal ganglion neurons using the MG132 induction method since the poor transfection efficiency in these precludes the use of α-syn constructs (Fig. 1Q–X). Differentiation was confirmed by staining for tyrosine hydroxylase (anti-TH1 antibody in green). Aggresomes could also be generated both types of cell when they were treated with MG132, as assayed by vimentin staining (Fig. 1Q,R,U,V) and γ-tubulin staining (Fig. 1S,T,W,X). Similarly, aggresomes were generated in immortalised human retinal pigment epithelial (RPE1-hTERT) cells and mouse embryonic fibroblast (MEFs) cells, which were used in subsequent experiments alongside SH-SY5Y cells (Fig. 1Y-β).

### Aggresomes suppress microtubule nucleation

**Fig. 2.**
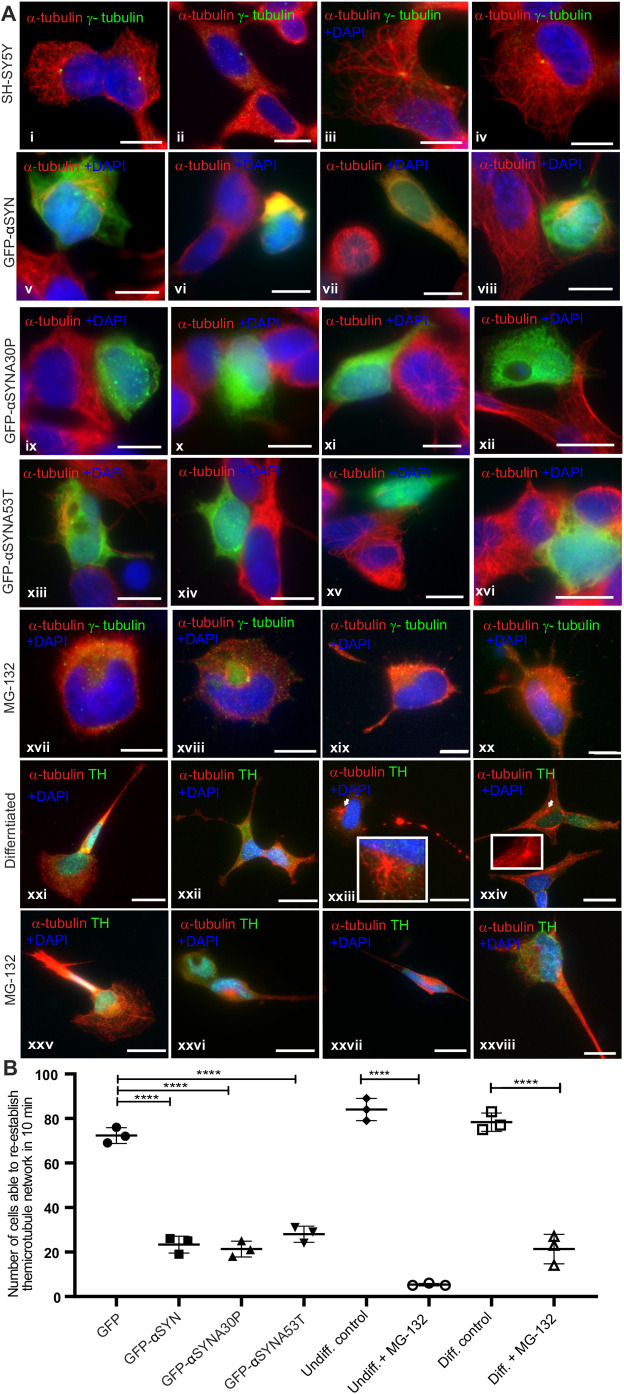
**Microtubule nucleation is disrupted in the presence of aggresomes** **in undifferentiated and differentiated SH-SY5Y cells**. (Ai–iv) SH-SY5Y cells have an extensive microtubule network. Upon cold treatment (4°C, *t*=0), microtubules depolymerise. Upon warming, microtubules nucleate from the centrosome forming a characteristic aster, which continues to grow until the network is re-established. In SH-SY5Y cells the aster is seen within in 30 s (37°C, *t*+0.5) and the microtubule network is re-established within 10 min (37°C, *t*+10). (Av–viii) In the presence of aggresomes formed by the overexpression of α-syn (GFP-fusion), the centrosome is unable to re-establish the network in 10 min. (Aix–xvi) The same result is achieved by overexpression of α-syn A30P and A53T familial mutants. (Axvii–xx) In the presence of aggresomes formed after MG132 treatment, the centrosome is unable to nucleate microtubules to re-establish this network. (Axxi–xxiv) In differentiated SH-SY5Y (tyrosine hydroxylase, TH, in green), microtubule nucleation is seen as asters from the centrosome (arrow heads and inset). (Axxv–xxviii) In the presence of aggresomes the density of the network is reduced and microtubule nucleation is severely compromised. (B) Quantification of microtubule regrowth in experiments in A [*P*=0.0001, one-way ANOVA (for α-syn over-expression), 100 cells, *n*=3 replicates; *P*=0.0001, by Student's *t*-test (MG132 experiments), 100 cells, *n*=3 replicates]. Microtubule nucleation and re-establishment of this network was quantified by scoring cells (yes or no) whether the network was re-established in 10 min. Microtubule staining for α-syn experiments shown separately in Fig. S1 together with GFP control. Scale bars: 10 μM.

The major function of the centrosome in interphase cells is the nucleation and organisation of the microtubule network (Bornens, 2002). The ability of the centrosome to nucleate microtubules can be assayed by the microtubule regrowth assay, in which the microtubules are first depolymerised by cold treatment for 30 min (Fig. 2Ai,ii) followed by warming of the cells so that the centrosome can nucleate a new network (Fig. 2Aiii, iv) (De Brabander et al., 1986; Fry et al., 1998). In control, untreated SH-SY5Y cells, the microtubules were nucleated after 30 s of warming following depolymerisation, with a clear aster of α-tubulin staining and an extensive network after 10 min (Fig. 2Ai–iv). For SH-SY5Y cells transfected with expression constructs for GFP-tagged α-syn, only 23±3.5% of the cells initiated microtubule nucleation by 10 min, whereas 72±3.8% of cells transfected with a control GFP expression plasmid re-established their network within the same time (Fig. 2Av–viii,B; Fig. S1). Cells transfected with α-syn with familial A30P or A53T mutations also failed to regrow their network within 10 min (Fig. 2Aix–xvi; Fig S1). Generating aggresomes via a second, independent route, gave the same result: cells treated with MG132 for 24 h prior to the assay did not nucleate any microtubules after 10 min warming (Fig. 2Axvii–xx), with only 5.3±0.58% of treated cells starting nucleation but 84±5.0% of untreated cells making an aster (Fig. 2B).

Due to the morphology of the cell and the number of stabilized microtubules, the network and reforming aster is more difficult to observe in differentiated SH-SY5Y cells than in continuously growing cell lines. Nevertheless, when aggresome formation was induced by MG132 treatment 21±6.7% of treated cells were able to make an aster, whereas 78±4.2% of untreated cells re-establish their labile microtubule network (Fig. 2Axxi–xxvii,B).

### Aggresomes prevent cell migration

**Fig. 3.**
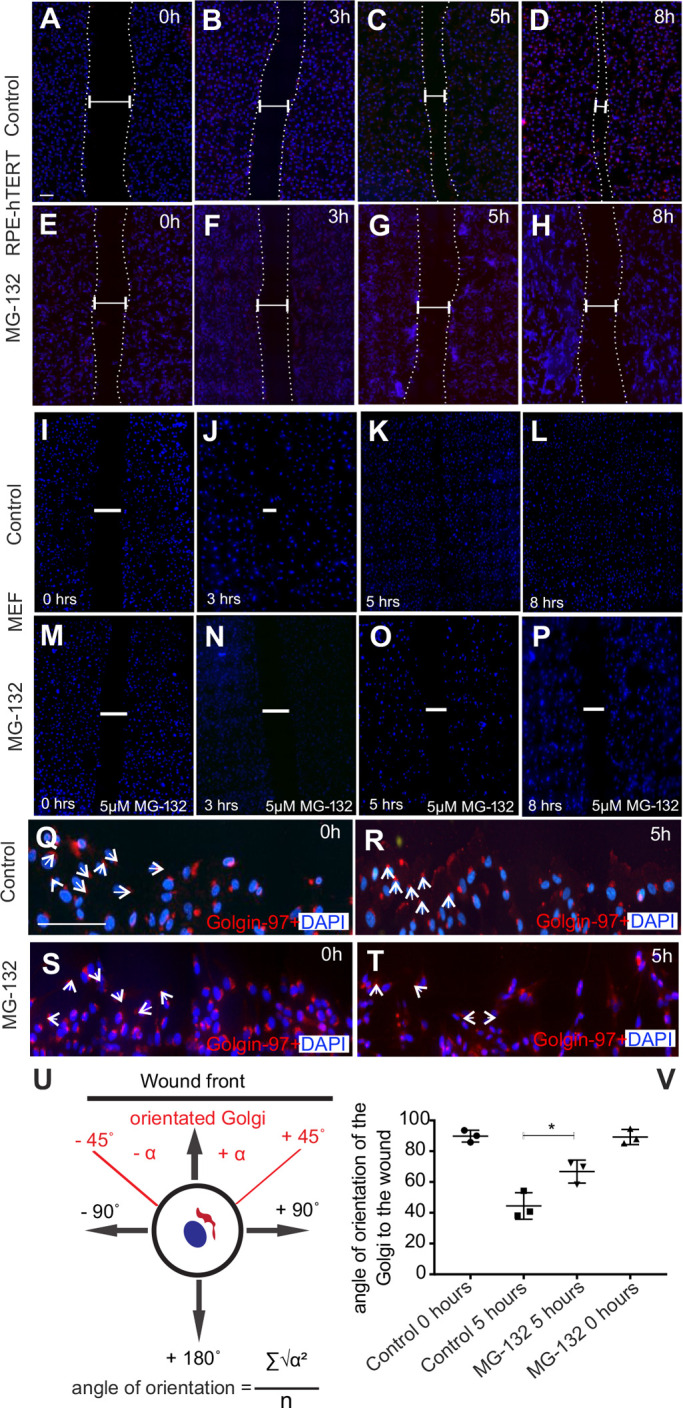
**Aggresomes reduce rate of cell migration and inhibit polarity changes.** (A–D) RPE1 cells close a scratch/wound in 8 h. (E–H) In the presence of aggresomes, minimal cell migration is detected. (I-L) MEFs close a scratch-wound assay in 8 h whereas those treated with MG132 to induce aggresomes fail to do so (M–P). (Q,R) In control cells, the Golgi (red) orientates from a random direction to face the leading edge of the wound. (S,T) In the presence of aggresomes, this change in orientation was not seen. (U,V) Quantification of change in angle of orientation during cell migration with a schematic diagram showing how the Golgi orientation was measured. (*P*=0.0011, by Student's *t*-test, 100 cells, *n*=3 replicates). Scale bars: 100 μM. Nuclei stained with DAPI (blue).

In healthy cells, the microtubule network is remodelled in response to polarity cues. This function of the centrosome can be tested by the wound assay. A strip of cells is removed from a confluent culture and those at the border of this wound will migrate to close the gap (Nobes and Hall, 1999). The Golgi and centrosome re-orientate to face the direction of cell locomotion. RPE1-hTERT cells and MEFs migrate and close a ‘wound’ in 12 h (Fig. 3A–D,I–L). In the presence of aggresomes generated by MG132 treatment, migration was halted with no cell movement to close the gap (Fig. 3E–H,M–P). Time-lapse observations of treated and untreated cells (Supplementary Movies 1–4) clearly demonstrate this lack of migration. 25% of the wound was closed in treated cells versus near-complete wound closure for untreated cells. The Golgi also did not re-orientate: control RPE1-hTERT cells’ Golgi re-orientated to face the wound (Fig. 3Q versus R), whereas in treated cells the Golgi remained randomly orientated (Fig. 3S versus T). If we measure the angle of the Golgi relative to a line perpendicular the wound, then control cells at 5 h face the wound (average of 44.2° displacement from perpendicular, where 45° displacement from perpendicular represents ‘perfect’ alignment) whereas treated cells at 5 h have a random orientation of the Golgi (average of 66.7° displacement from perpendicular) (Fig. 3U,V).

### Aggresome formation inhibits ciliogenesis

**Fig. 4.**
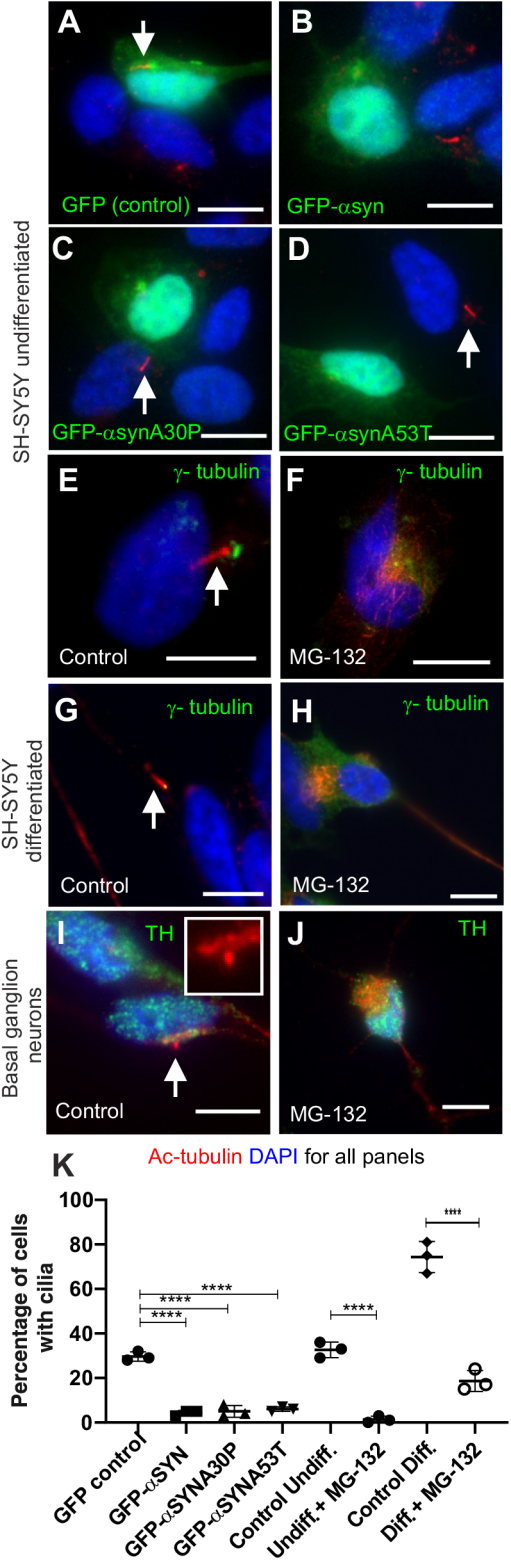
**Aggresomes inhibit ciliogenesis.** All panels: DNA/nuclei stained with DAPI (blue), green label is indicated in each panel, cilia (white arrows) can be identified by acetylated tubulin (red). (A–F) When transfected with a GFP-α-syn expression plasmid (α-syn and A30P and A53T familial mutants) or treated with MG132, cilia formation is inhibited in undifferentiated SH-SH5Y cells (B, C and D versus A, F versus E). In the presence of aggresomes differentiated SH-SY5Y cells are no longer able to form cilia (H versus G). When treated with MG132, TH-positive basal ganglion neurons are no longer able to form cilia (J versus I). The acetylated tubulin signal (red) in J and I is overexposed to ensure no cilia were missed. (K) Quantification of ciliation: GFP expressing versus GFP-α-syn expression, (*P*=0.0003, by one-way ANOVA, 100 cells, *n*=3); undifferentiated SH-SY5Y, untreated versus MG132, (*P*=0.0001, by Student's *t*-test, 100 cell, *n*=3); differentiated SH-SY5Y, untreated versus MG132, (*P*=0.0001 by Student's *t*-test, 100 cells counted, *n*=3). Scale bars: 10 μM.

We next tested if the presence of aggresomes prevents cells from making cilia as ciliogenesis requires the centrioles of the centrosome to move to the cell surface where one centriole templates the axoneme that forms the internal structure of the cilium. Many cell types ciliate, but not all. We tested several cell types for the presence of cilia by staining for acetylated tubulin together with γ-tubulin to mark the basal bodies. Undifferentiated SH-SY5Y cells generated cilia when serum starved and GFP over-expression (control plasmid) did not affect ciliogenesis (Fig. 4A,E,K). Undifferentiated SH-SY5Y cells failed to ciliate when GFP-α-syn (Fig. 4B) was overexpressed. Familial mutant forms of GFP-α-syn – A30P and A53T – had the same effect (Fig. 4C,D,K). MG132 treatment also reduced ciliogenesis (Fig. 4F,K).

Differentiated SH-SY5Y cells formed cilia (Fig. 4G) but did not do so when treated with MG132 (Fig. 4H,K). Rat basal ganglion cells are ciliated under their normal culture conditions (Fig. 4I) but their ciliogenesis was much reduced when treated with MG132 (Fig. 4J; Fig. S2).

**Fig. 5.**
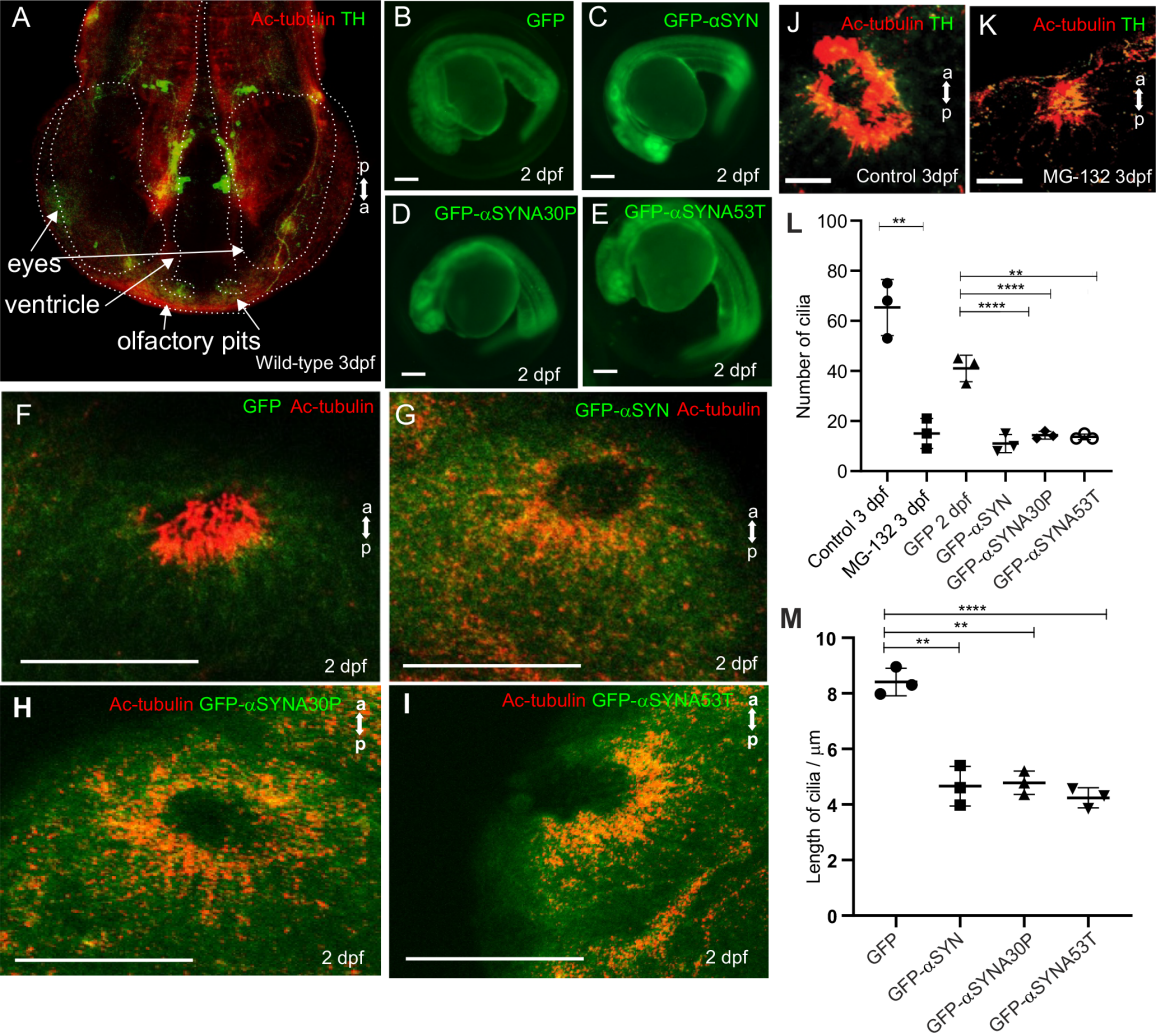
**Olfactory cilia in zebrafish larvae are severely reduced in the presence of aggresomes.** (A) The neuronal dopaminergic network in 3 dpf zebrafish forebrain viewed from the dorsal aspect, detected by TH-staining (green). Acetylated tubulin (red) stains axon tracts and cilia. (B–E) Overexpression of control GFP or α-syn, α-synA30P, α-synA53T familial mutations does not cause any anatomical defects in zebrafish larvae. (F) By 3 dpf extensive numbers of cilia are visible at the olfactory pit. (G–I) Overexpression of any of the three forms of α-syn (wild type, A30P or A53T) severely reduces numbers of cilia in the olfactory pit. Cilia length is also reduced. (J,K) Embryos treated with MG132 showed an extensive reduction in number of cilia. (L) Quantification of cilia numbers from above experiments (*P*=0.001, by one-way ANOVA, *n*=3). Cilia were counted from the confocal generated z-stacks, projected images of which are shown in this figure. (M) Quantification of length of cilia from F-I (*P*=0.0088, by one-way ANOVA, *n*=3). Scale bars: 100 μM.

To test whether aggresome formation affected ciliogenesis in an *in vivo* system, we performed α-syn over-expression experiments in zebrafish embryos. The ciliated cells of the olfactory pit are dopaminergic neurons, as indicated by tyrosine hydroxylase (TH)-positive staining (Fig. 5A). We injected 1-cell embryos with *in vitro* transcribed mRNA encoding human α-syn, (wild-type and familial A30P and A53T mutations) or GFP as a control (Fig. 5B–E). Larvae were fixed at 2 days post-fertilisation (dpf) and assayed for olfactory cilia by wholemount acetylated tubulin staining (Fig. 5F–I). Cilia numbers were much reduced (11±3.6 versus 41±5.3 per pit) in the olfactory zone when the embryos overexpressed GFP-α-syn (any variant) compared to GFP alone (Fig. 5L). The remaining cilia were reduced in length, by approximately 50% from 8.4±0.49 µm to 4.7±0.71 µm (Fig. 5M). Treatment of embryos with MG132 caused loss of cilia at 3 dpf (Fig. 5J, untreated; K, treated) with cilia number being reduced from 65±11 per pit to 15±6 (Fig. 5L). Although we did not attempt to image aggresomes in these embryos, the difference between treated and untreated embryos, achieved by two different methods, strongly suggests the effects are due to the presence of aggresomes. The gross anatomy of these embryos was otherwise normal (Fig. 5B–E). Absence of cilia may be expected to cause other defects during embryogenesis, for example hydrocephaly, left–right asymmetry defects and pronephric cysts. We did not observe these defects. However, the effect on ciliogenesis at the olfactory pits was mild at 24 hpf. We therefore suspect that accumulation of α-syn is required over several days to inhibit ciliogenesis hence earlier embryonic stages escape the effects of aggresome-induced cilium loss. Similar loss of cilia was observed when aggresomes were generated by an independent method, MG132 treatment (Fig. 5J–L).

## DISCUSSION

Aggresomes occupy a central position in the cell, at the hub of the microtubule network, a location that places them in the vicinity of the centrosome, the major microtubule organising centre of the cell. Furthermore, the aggresome acquires an important centrosomal component, γ-tubulin. We show here that α-syn accumulation is sufficient to form aggresomes and these structures severely compromise centrosome function. Microtubule nucleation is severely reduced and the centrosome is unable to be repositioned during the scratch-wound assay. These results are consistent with and extend previous observations of the effect of MG132 on microtubule nucleation (Didier et al., 2008; Diaz-Corrales et al., 2012). A simple, steric hindrance of the centrosome is the simplest and most likely explanation for this effect but it is also possible mislocalisation or modification of specific centrosome proteins could also cause the observed effects. For example, the accumulation of γ-tubulin at the aggresome may sequester γ-tubulin molecules intended for the centrosome. Importantly, we also observe these effects when generating aggresomes by overexpressing α-syn. Indeed, MG132 treatment generates aggresomes in which endogenous α-syn is accumulated. This suggests the effects are due to the aggresome itself rather than inhibition of the proteasome and consequent modulation of other pathways. Our data suggests that induction of aggresomes via α-syn over-expression and via MG132 treatment produces functionally equivalent aggresomes and these findings help to unify the literature and observations made in previous studies using only MG132 induction. It is important to note that reduction in proteasome function is physiologically relevant in the context of neurodegenerative disease. Indeed, deficits in the ubiquitin-proteasome degradation system are well-established as causative factors in ageing and neurodegenerative diseases (for review see Rousseau and Bertolotti, 2018) and several recent studies have demonstrated that GWAS-identified variants associated with a range of neurodegenerative diseases cause deficits in proteasome function (Lopez et al., 2017; Jabbari et al., 2018).

The centrosome is the main microtubule organising centre in the cell, nucleating microtubules and anchoring a portion near to the nucleus. Its role as a major component of the spindle poles is not important in mature neurons, which do not divide. However, a functioning, polarised microtubule network is still required for intracellular trafficking in terminally differentiated cells such as neurons and the centrosome is essential for maintaining this.

The inability of cells to migrate and the Golgi to re-orientate in the scratch-wound assay provides a useful surrogate read-out for the inability to repolarise the microtubule network in a desired direction. In mature neurons, compromised cell polarity could have severe long-term effects on neuronal function and survival. A polarised microtubule network is essential for the rapid trafficking of organelles and vesicles, especially those containing neurotransmitters that need to be transported to synapses, and the recycling of materials involved in neurotransmission. Diaz-Corrales et al. also observed effects on intracellular transport of the dopamine receptor, due to fragmentation of the Golgi (Diaz-Corrales et al., 2012). Our data suggest that disruption of cell polarity could also contribute to this impaired transport.

Our results show that aggresomes can prevent a cell from turning its centrosome into a cilium. Proteasome inhibition is known to inhibit ciliogenesis by preventing proteasomal degradation of ciliogenesis inhibitory proteins such as trichoplein (Kasahara et al., 2014). Our data, in which aggresomes are also generated by α-syn overexpression, not just proteasome inhibition, point to the aggresome inhibiting this process by itself. Whether this is a steric effect or due to competitive inhibition of degradation of trichoplein and similar molecules remains to be determined. In PD, this may be relevant to those cells that undergo ciliogenesis. An early symptom of PD is loss of smell and anosmia may precede other symptoms (Doty et al., 1988; Haehner et al., 2007). It is not clear what the cause of anosmia is, the assumption being that loss or impairment of neurons processing olfactory information is the cause. An alternative explanation is that the olfactory neurons themselves are affected during PD progression. Olfactory neurons are part of the dopaminergic system (Pignatelli and Belluzzi, 2017) and the olfactory receptors are housed in cilia on these cells. If early in the development of PD the ability of olfactory neurons to renew cilia was compromised then the sense of smell would be affected as a consequence. This hypothesis certainly warrants further investigation as it may reveal that the olfactory cells are the sentinels of the dopaminergic system and anosmia represents a first indicator of dopaminergic cell dysfunction. While proving that Lewy bodies affect microtubule nucleation and cellular polarity in neurons of PD patients is likely to prove difficult, nasal brushing, a method to sample olfactory neurons from the epithelium (Orru et al., 2014), might offer a route to analyse the olfactory cilia. Indeed, this process has been able to detect α-syn and other aggregating proteins involved in neurodegenerative diseases in olfactory neurons from patients (Brozzetti et al., 2020).

## MATERIALS AND METHODS

### Cell culture

Cell lines used include: HeLa, provided by Prof. George Dickson at Royal Holloway; neuroblastoma cell line SH-SY5Y, provided by Prof. Robin Williams at Royal Holloway; RPE1-hTERT, kindly provided by Prof. Erich Nigg, Basel, Switzerland; MEFs, provided by Dr Jenny Murdoch at Royal Holloway. Primary rat basal ganglion neurons were prepared by Dr Simona Ursu at Royal Holloway.

HeLa and SH-SY5Y cells were maintained in Dulbecco's Modified Eagle's Medium (DMEM) supplemented with 10% Foetal Bovine Serum (FBS), 1x antibiotic-antimycotic mix (Gibco; 5000 units of penicillin, 5000 μg streptomycin and 15 μg Amphotericin B) and 2 mM L-glutamine. RPE1-hTERT were grown in DMEM with nutrient mixture F-12 Ham (DMEMF-12) supplemented with 10% FBS, 1× antibiotic-antimycotic (5000 units of penicillin, 5000 μg streptomycin and 15 μg Gibco Amphotericin B), 2 mM L-glutamine and 0.38% sodium bicarbonate. MEFs were grown in DMEM supplemented with 10% FBS, 1× antibiotic-antimycotic, 2 mM L-glutamine and 1x non-essential amino acids. All cell lines were cultured at 37°C in a humidified atmosphere with 5% CO_2_. The same media mix was used for future experimental assays unless otherwise stated.

### Primary basal ganglion neurons

Primary cultures of basal ganglion neurons were prepared from E18 Sprague Dawley rat embryos as previously described (Marsh et al., 2017). Briefly, cells were plated at a density of either 75,000 or 500,000 cells on poly-d-lysine-coated (Sigma-Aldrich, 0.1 mg/ml in borate buffer pH 8.5) 22 mm^2^ glass cover slips. The plating medium was DMEM supplemented with 5% FBS, penicillin/streptomycin and 0.5 mM l-glutamine (all from Invitrogen). On the next day the medium was changed to full neurobasal medium (neurobasal medium supplemented with B27, 0.5 mM l-glutamine, all from Invitrogen). Cultures were incubated at 37°C and 5% CO_2_, and were used at 18 days *in vitro*.

### SH-SY5Y differentiation

Cells were plated on collagen-coated coverslips in 12-well plates. Optimised seeding density was calculated to be 6×10^4^ cells in 12-well culture plates. Cells were seeded out in DMEM serum supplemented media, The media was replaced the following day with DMEM-F12 supplemented with 1% FBS, 2 mM L-glutamine, 1× antimicrobial mix (5000 units of penicillin, 5000 μg streptomycin and 15 μg Gibco Amphotericin B), 1× non-essential amino acids and 10 μM all trans-retinoic acid (RA). Every 2 days the media was replenished and after 7 days differentiation was observed.

### Aggresome formation

Aggresomes were induced by either treating cells with the proteasome inhibitor MG132 (Sigma-Aldrich) or by overexpressing GFP-tagged human α-syn. The optimal MG132 concentration was determined for each cell line ranging from 1 μM to 10 μM (HeLa, 10 μM; SH-SY5Y, 1 μM; differentiated SH-SY5Y, 1 μM; and rat neurons, 3 μM). MG132 was added to media for 18 h at 37°C in a humidified atmosphere with 5% CO_2_.

Constructs including pEGFP-C2, pEGFP-αSYN, pEGFP-αSYNA30P and pEGFP-αSYNA53T were transiently transfected into cells. Lipofectamine 2000 (Thermo Fisher Scientific) was used as the transfection reagent following the manufacturer's instructions. In brief, cells were plated onto glass coverslips in serum-supplemented media without antibiotic-antimycotic mix. At 70% confluency cells were transfected with DNA-lipid complexes (2.5 μg of plasmid DNA was diluted in 250 µL Opti-MEM+10 μL of Lipofectamine 2000 reagent in 240 μL of Opti-MEM). Complexes were added to each well for 5 h at 37°C after which the media was replaced by low serum-supplemented media (DMEM, 1% FBS, 2 mM L-glutamine and 1% antibiotic-antimitotic mix for HeLa and SH-SY5Y; DMEM-F12, 1% FBS, 1% antibiotic-antimitotic mix for RPE1-hTERT). Overexpression was achieved at 72 h from the time of transfection.

### Microtubule re-growth assay

Cells were plated onto ethanol-washed glass coverslips in serum-supplemented media. At 70% confluency the samples were processed for the respective condition (MG132 treatment or overexpression of α-syn). The plate was incubated on ice for 30 min then pre-warmed media (37°C) was added. Samples were fixed at different time points ranging from 0.5 min to 25 min, depending on the cell line, including immediately after 30 min on ice. Cells were fixed for 10 min with methanol (−20°C), washed with PBS and stored in PBS.

### Ciliogenesis assay

Cells were plated on glass coverslips in 6- or 12-well culture plates in serum-supplemented media. Cells were treated with MG132 or transfected with α-syn overexpression constructs to form aggresomes. The media was replaced the following day with serum free media; samples were incubated for 24 h to induce cilia formation. Cells were fixed using 4% (v/v) formaldehyde (FA) for 10 min. FA was aspirated and cells were washed with PBS. Samples were stored in 0.2% Triton in PBS until they were processed for immunocytochemistry.

### Cells migration assay (scratch-wound assay)

MEFs and RPE1-hTERT were used in the wound assay. MEFs were plated onto collagen-coated 35 mm plastic Petri dishes. At 100% confluency (with or without aggresome formation using MG132) a P200 pipetman tip was used to make wound. The media was aspirated and washed twice with 1× PBS to remove detached cells. CO_2_ independent media supplemented with 10% FBS, 2 mM L-glutamine, 1× antibiotic-antimycotic mix was added for time-lapse experiments. Images were taken using a Nikon TE300 microscope with a 37°C chamber, over a 24-h period with images taken every 2 min. Similarly, RPE1-hTERT cells were plated onto ethanol-washed glass coverslips and treated with MG132. Cell migration was assessed by fixing cells at set time points with ice-cold methanol.

### Golgi orientation

A Golgi positioned within −45° and +45° of the wound was considered to be orientated towards the wound. The average angle of orientation was calculated using the formula (Σ√*α*^2^)/*n* where *α* is the angle between the Golgi and a line perpendicular to the wound edge.

### Immunocytochemistry

Cells were fixed with either methanol (−20°C) or 4% (v/v) FA for 10 min. The fixative was removed and washed with PBS (3×5 min). Cells were blocked in 3% BSA for 30 min at room temperature. Cells were incubated with primary antibody in 1% BSA, either for 3 h at room temperature on a shaking platform or overnight at 4°C on a shaking platform. After primary antibody incubation the cells were washed with PBS (3×5 min) and incubated with secondary antibody for 1 h at room temperature on a shaking platform. Cells were washed with PBS (3×5 min) and mounted using FluorSave (Calbiochem). Primary antibodies used were: rabbit anti-Tyrosine Hydroxylase (Merck-Millipore), 0.1 μg/ml; mouse anti-vimentin (Sigma-Aldrich), 1 μg/ml; mouse anti-acetylated-tubulin (Invitrogen) 1 μg/ml; mouse anti-γ-tubulin (Sigma-Aldrich), 1 μg/ml; rabbit anti-γ-tubulin (Sigma-Aldrich), 1 μg/m; mouse anti-α-tubulin (Sigma-Aldrich), 1 μg/ml; rabbit anti-α-syn (Cell Signaling Technology), 10 μg/ml; mouse anti-Golgin 97 (Thermo Fisher Scientific), 1 μg/ml. Secondary antibodies used were: goat anti-mouse IgG, Alexa Fluor 594-conjugated (Invitrogen) 1 μg/ml; goat anti-mouse IgG, Alexa Fluor 488-conjugated (Invitrogen) 1 μg/ml; and goat anti-rabbit IgG Alexa Fluor 488-conjugated (Invitrogen) 1 μg/ml. Images were taken using a Nikon Ni-E fluorescence microscope.

### Wholemount immunostaining

Embryos were fixed with Dent's fixative (80:20, methanol: DMSO) or 4% (v/v) FA overnight at 4°C. Fixative was removed the following day; embryos fixed with Dent's fixative were stored in methanol at −20°C; FA fixed embryos were stored in PBS +0.2% Triton. Embryos fixed with Dent's fixative were permeabilised by incubation in 100% methanol for 30 min at −20°C. Embryos were rehydrated by washing in serial dilution of methanol in PBS including: MeOH:PBS at 70:30, 50:50 and 30:70 and final wash with PBS. FA fixed embryos were permeabilised by incubation in 0.25% trypsin-EDTA in PBS for 10 min on ice and then washed three times for 30 min in PBS +0.02% Triton. Embryos were blocked for 4 h in 10% heat-inactivated goat serum, 1% bovine serum albumin and 0.2% Triton in PBS. Embryos were incubated with primary and secondary antibodies for 36 h in blocking solution. Primary antibodies used: rabbit anti-Tyrosine Hydroxylase (MERK Millipore), 0.1 μg/ml; and mouse anti-acetylated-tubulin (Invitrogen) 1 μg/ml. Secondary antibodies used were goat anti-mouse IgG, Alexa Fluor 594-conjugated (Invitrogen) 1 μg/ml; and goat anti-rabbit IgG Alexa Fluor 488-conjugated (Invitrogen) 1 μg/ml. Confocal stacks were imaged with an Olympus FX81/FV1000 laser confocal system using Ar gas laser and He-Ne diode laser. Stacks were taken in 1 μM thickness and are represented as maximum-intensity projections. Stacks were analysed using ImageJ.

### mRNA synthesis

mRNAs were transcribed from the Sp6 promoter of the pCS2+-based plasmids encoding α-syn and mutant forms, using the mMessage mMachine *in vitro* transcription kit (Ambion, TX, USA). RNA was purified using the Qiagen RNeasy kit (Qiagen).

### Zebrafish

Zebrafish were maintained and bred at 26.5°C; embryos were raised at 28.5°C. Both AB and TL wild-type strains were used for these studies. Embryos were processed by 33 dpf. No protected species, as defined by the Animals (Scientific Procedures) Act, 1986 were used for experiments in this study. Embryos were injected into the yolk with mRNA using a micromanipulator-mounted micropipette (Borosil 1.0×0.5 mm, Frederick Haer & Co., Inc., USA) and a Picospritzer microinjector. Between 150–200 pg of mRNA was injected into the yolk of the embryos at 1–4 cell stage. For MG132 treatment, embryos were treated with 50 μM for 48 h. Embryos processed for immunostaining were grown in 0.003% phenylthiourea to inhibit melanin production.

### Statistics

Statistical tests used for each experiment are given in the figure legends. *t*-tests were used when comparing two treatments and ANOVA for multiple treatments. *n* numbers represent the experimental unit, either number of embryos or separate cell culture experiments. Error bars show the standard deviation. Analysis performed using GraphPad Prism v8.

### Animal studies

No protected stages of zebrafish, as defined by the Animals (Scientific Procedures) Act 1986, were used for experiments in this study, only fry less than 5 dpf. Rats were killed by Schedule 1 methods, according to Home Office regulations, in compliance with the Animals (Scientific Procedures) Act 1986.

## Supplementary Material

Supplementary information
