# Supplementary figures and images for: Alpha-synuclein aggresomes inhibit ciliogenesis and multiple functions of the centrosome
Source: Biol Open. 2020 Oct 5;9(10):bio054338. doi: 10.1242/bio.054338 (PMC7561473; doi:10.1242/bio.054338)

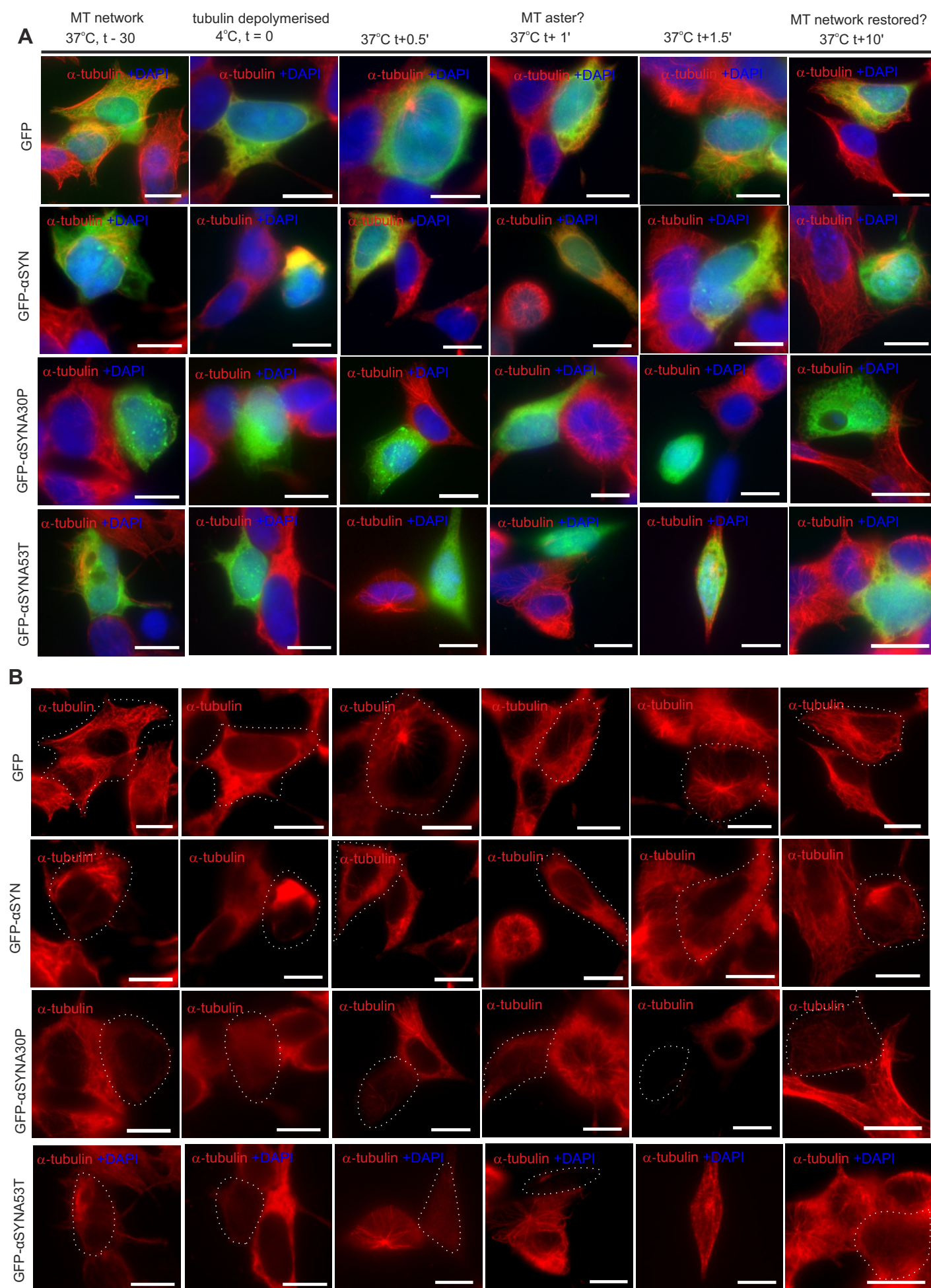

Fig S1

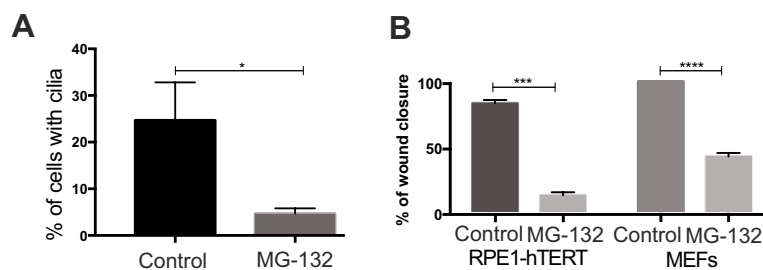

**Fig S2**

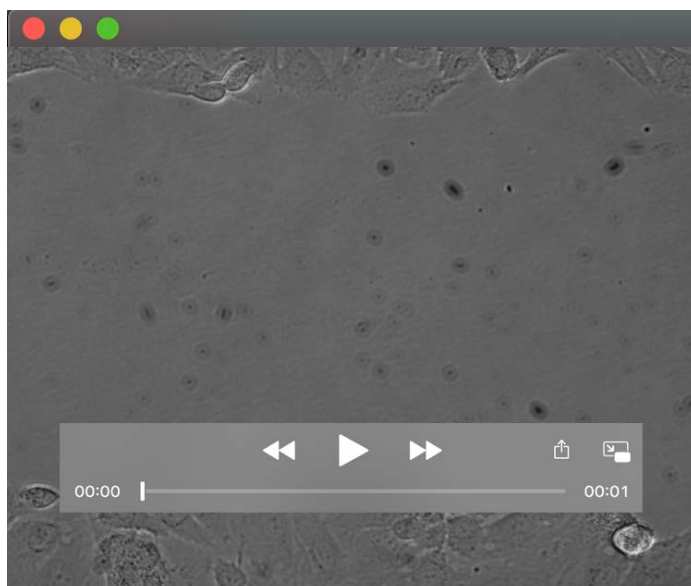

Movie 1. RPE control

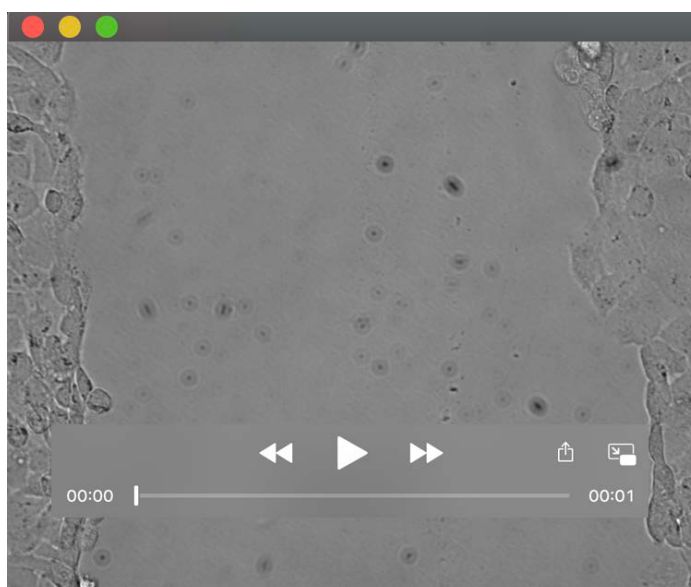

Movie 2. RPE MG132

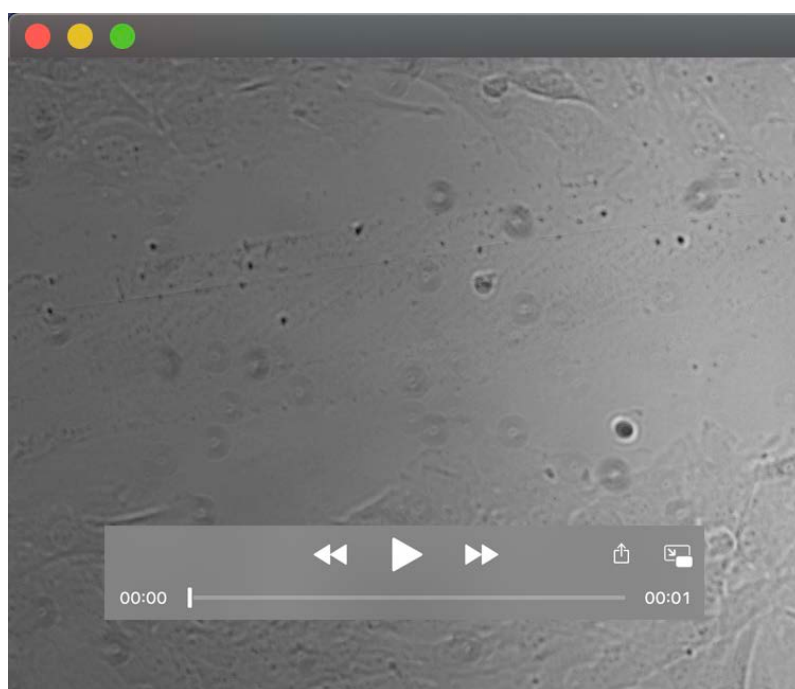

Movie 3. MEF control

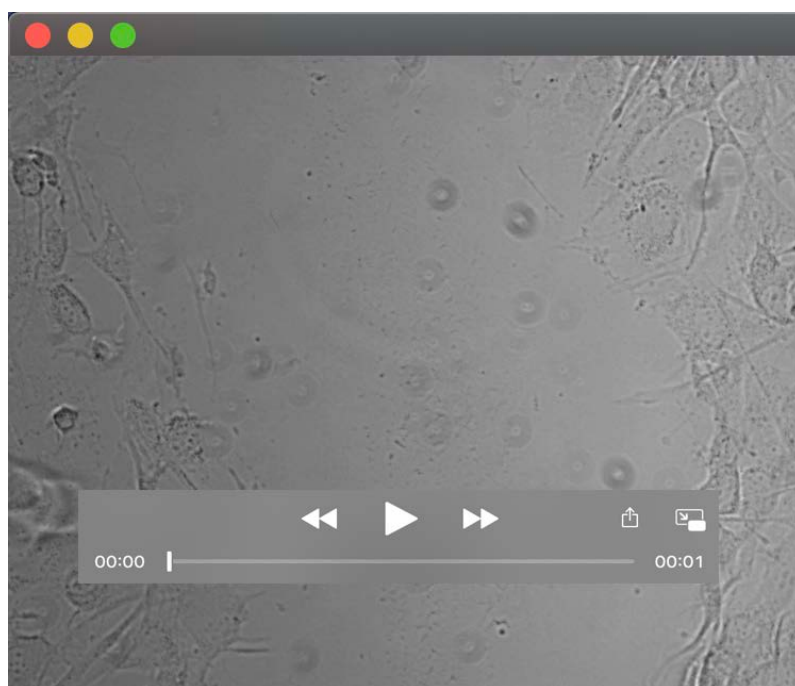

Movie 4. MEF MG132

Supplement: Supplementary information [file biolopen-9-054338-s1.pdf]
